# Polypropylene Mesh-Augmented Soft-Tissue Reconstruction for Presumed Traumatic Lateral Patellar Luxation in an Adult Pony: A Case Report

**DOI:** 10.3390/ani16213501

**Published:** 2026-11-05

**Authors:** Sihyeon Hwang, Geonil Moon, Young-Gwang Cho, Chanil Park, Inhyung Lee, Tran Thi Kim Ngan, Namsoo Kim

**Affiliations:** 1 Department of Veterinary Surgery, College of Veterinary Medicine, Jeonbuk National University, Iksan 54596, Republic of Korea; ghkddlsrl95@jbnu.ac.kr (S.H.); 202450305@jbnu.ac.kr (G.M.); 2 Department of Veterinary Clinical Sciences, College of Veterinary Medicine, Seoul National University, Seoul 08826, Republic of Korea; beglory@snu.ac.kr (Y.-G.C.); cksdlf342@snu.ac.kr (C.P.); inhyunglee@snu.ac.kr (I.L.); 3 School of Agriculture and Natural Resources, Vinh University, Vinh City 43131, Vietnam; nganttk@vinhuni.edu.vn

**Keywords:** equine, pony, lateral patellar luxation, polypropylene mesh, soft-tissue reconstruction, stifle, trauma

## Abstract

Patellar luxation is uncommon in horses, and traumatic lateral luxation in adult equids has rarely been reported. A 3-year-old, 75 kg female Shetland pony presented with severe right hindlimb lameness and minimal weight bearing approximately 2 weeks after a suspected traumatic event. The right patella was fixed lateral to the femoral trochlea and could not be manually reduced during conscious examination. Radiographs confirmed lateral patellar luxation without apparent osseous deformity. Following general anesthesia and surgical exposure, the patella was manually reduced. Treatment comprised lateral retinacular and fascial release, medial capsular imbrication and extra-articular polypropylene mesh augmentation. No osseous corrective procedure was performed. Radiographs obtained 2 days postoperatively confirmed maintenance of reduction. Partial weight bearing was observed approximately 10 days after surgery. No surgical site complications were observed during hospitalization, and the pony was discharged on postoperative day 12. At telephone follow-up 37 days after surgery, the owner reported that the pony was walking more frequently. An owner-provided video recorded on postoperative day 130 showed no overt lameness during walking; trotting was not assessed, and no further clinical examination or radiography was performed. Mesh-augmented soft-tissue reconstruction was technically feasible in this adult pony, although objective follow-up remained limited.

## Simple Summary

Patellar luxation, in which the kneecap moves out of its normal position, is uncommon in horses. This report describes a 3-year-old Shetland pony with severe right hindlimb lameness after a suspected traumatic event. The kneecap was fixed on the outer side of the joint and could not be repositioned while the pony was awake. Radiographs showed no obvious bone deformity. During surgery, the kneecap was repositioned, surrounding tissues were adjusted, and a synthetic polypropylene mesh was added for reinforcement. Radiographs obtained 2 days after surgery confirmed that the kneecap remained in position. The pony began to bear some weight on the operated limb, and the owner reported more frequent walking 37 days after surgery. An owner-provided video recorded 130 days after surgery showed no obvious limping during walking. However, no further examination or radiographs were available; so, the position of the kneecap and complete recovery could not be confirmed.

## 1. Introduction

Patellar luxation is an uncommon orthopedic disorder in horses and may be congenital, developmental, or traumatic in origin [1,2]. Lateral displacement is the most frequently described form in equids [1]. Surgical management depends on the underlying abnormalities and may involve soft-tissue balancing, osseous reconstruction, or a combination of both [1,2]. Reported procedures include lateral soft-tissue release, medial capsular or retinacular imbrication, trochleoplasty, and tibial tuberosity transposition [1,2].

Traumatic lateral patellar luxation has been described in neonatal foals with associated medial periarticular soft-tissue injury [3,4]. These reports provide relevant comparisons for surgical management, although their applicability to adult ponies remains uncertain.

Polypropylene mesh reinforcement has been used as an adjunct to medial soft-tissue reconstruction [1,5]. Gustafsson et al. [5] placed mesh over imbricated medial tissues in an Arabian filly with congenital lateral patellar luxation. More recently, mesh augmentation combined with medial imbrication and block-recession trochleoplasty was described in a foal with bilateral lateral patellar luxation [1].

The present report describes polypropylene mesh-augmented medial soft-tissue reconstruction in a 3-year-old Shetland pony with a fixed lateral patellar luxation of presumed traumatic origin and no apparent osseous deformity. The surgical strategy and postoperative outcome are discussed in relation to previous traumatic and mesh-reinforced repairs [3,5].

## 2. Detailed Case Description

### 2.1. Case History and Clinical Examination

A 3-year-old, 75 kg female Shetland pony was presented on 15 June 2026 for severe right hindlimb lameness and minimal weight bearing. A traumatic event was suspected to have occurred during the evening of 31 May 2026, although it was not witnessed. The pony was found the following morning with near non-weight-bearing lameness of the right hindlimb and marked difficulty walking. Loose ropes were present in the surrounding area, and the pasture contained sloping grassland; therefore, entanglement or slipping was considered possible. The clinical signs persisted until referral approximately 2 weeks later. According to the owner, no gait abnormalities had been observed before this episode. No medication, prescribed rest or exercise restriction, bandaging, or attempted reduction had been undertaken before referral.

At presentation, the right patella was palpable lateral to the femoral trochlea and was fixed in this position. At rest, the right stifle was held in slight flexion, with only the toe of the right hind hoof contacting the ground and minimal weight bearing on the affected limb. Manual reduction during the conscious orthopedic examination was unsuccessful. The contralateral left stifle showed no apparent swelling, and normal weight bearing was observed on the left hindlimb. No clinically relevant abnormalities of the left stifle were identified. Ultrasonographic examination was not performed. Review of the preoperative gait video (Video S1) did not reveal an obvious functional abnormality of the left hindlimb. A specific assessment of craniocaudal femorotibial instability was not performed.

### 2.2. Diagnostic Imaging

**Figure 1:**
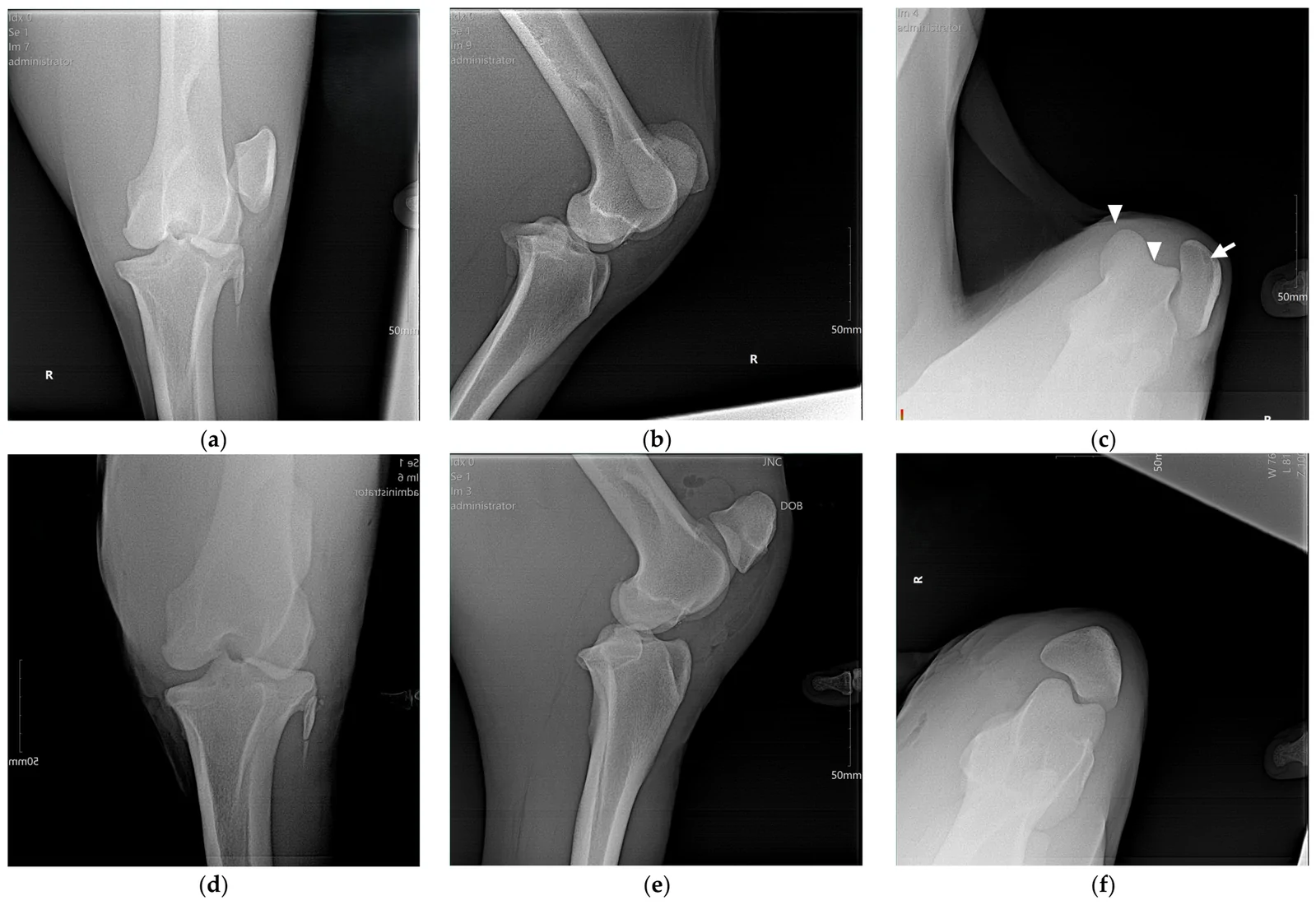
Preoperative and postoperative radiographic evaluation of the right stifle. (**a**–**c**) Preoperative caudocranial, lateromedial, and skyline views demonstrating lateral displacement of the patella relative to the femoral trochlea. (**d**–**f**) Corresponding views obtained 2 days after surgery demonstrating maintenance of patellar reduction. In (**c**), the white arrow indicates the patella, and the white arrowheads indicate the trochlear ridges. **R** indicates the right hindlimb.

Preoperative caudocranial, lateromedial, and skyline radiographic views of the right stifle demonstrated lateral displacement of the patella relative to the femoral trochlea (Figure 1a–c). No concurrent fracture or gross trochlear hypoplasia or deformity was identified. Assessment of trochlear morphology was qualitative; trochlear ridge height, symmetry, and sulcus depth were not quantitatively measured. On review of the preoperative lateromedial radiograph, the distal femur appeared slightly cranially positioned relative to the tibia (Figure 1b). Differences in stifle flexion and radiographic projection between the preoperative and postoperative images limited direct comparison of femorotibial alignment. Based on the acute onset of clinical signs and the environmental circumstances, a traumatic origin was suspected. However, the cause could not be confirmed, and the luxation was classified as being of presumed traumatic origin. Radiographs of the contralateral left stifle were not obtained.

### 2.3. Anesthesia and Surgical Treatment

Surgery was performed on 15 June 2026. The pony was premedicated with intravenous (IV) detomidine at 2 μg/kg. General anesthesia was induced with diazepam (0.1 mg/kg, IV) and ketamine (2.2 mg/kg, IV). An additional dose of ketamine (0.7 mg/kg, IV) was administered before endotracheal intubation. Following endotracheal intubation, anesthesia was maintained with isoflurane and mechanical ventilation. Procaine penicillin (14,000 IU/kg) and flunixin meglumine (1.1 mg/kg) were administered intramuscularly (IM) during the perioperative period. Dobutamine was administered as a continuous rate infusion at 1–4 μg/kg/min as required for cardiovascular support during anesthesia.

**Figure 2:**
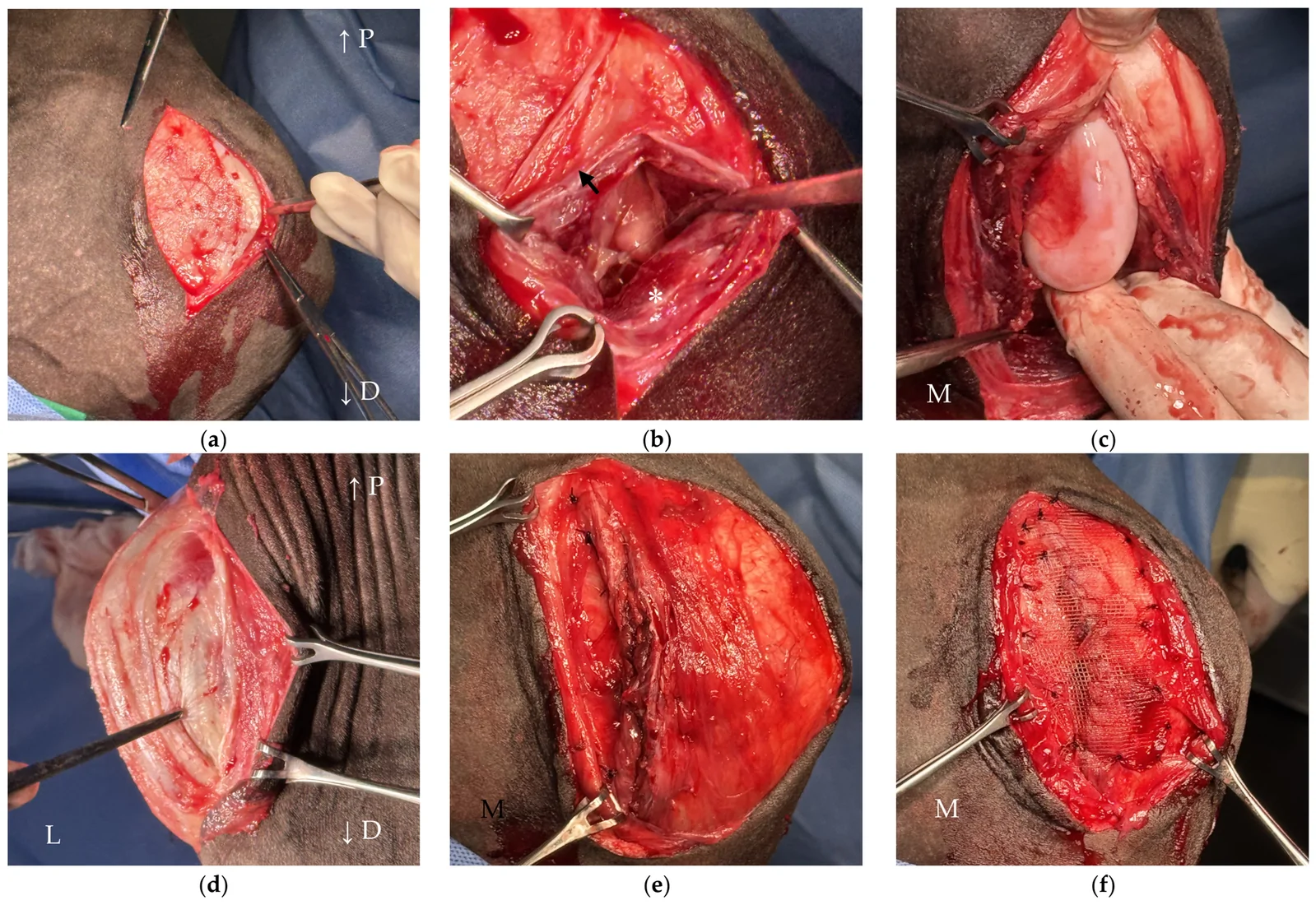
Intraoperative sequence of correction of right lateral patellar luxation. (**a**) Medial approach to the stifle. (**b**) Exposure of the femoropatellar joint following incision of the medial joint capsule. The sartorius muscle grasped with the forceps is indicated by a white asterisk. The black arrow indicates the visible incised margins of the medial fascia. (**c**) Gross appearance of the exposed femoral trochlea, with no apparent cartilage erosion in the visible area. (**d**) Lateral retinacular and fascial release through the medial approach with lateral retraction of the skin. (**e**) Medial capsular imbrication. (**f**) Extra-articular polypropylene mesh augmentation over the medial capsular repair. Orientation markers indicate proximal (P), distal (D), medial (M), and lateral (L) directions where applicable.

The pony was positioned in dorsal recumbency, and the right hindlimb was aseptically prepared and draped. A skin incision was made over the medial aspect of the right stifle. Following dissection of the subcutaneous tissues, the medial joint capsule was incised to expose the femoropatellar joint (Figure 2a–c).

Although manual reduction had been unsuccessful during the conscious examination, the patella could be manually reduced into the femoral trochlear groove following induction of general anesthesia and surgical exposure, before lateral release. Inspection of the exposed trochlea revealed no gross evidence of trochlear hypoplasia or morphological deformity. No gross traumatic disruption of the medial periarticular soft tissues was identified. After reduction and before lateral release, repeated flexion and extension of the stifle did not result in reluxation. Gross inspection of the patellar articular cartilage and the cartilage of the lateral trochlear ridge revealed no apparent abnormalities; dedicated photographs documenting these assessments were not obtained.

After reduction, the skin was retracted laterally through the existing medial approach to expose the lateral retinaculum and fascia. These structures were incised to perform the lateral release; no separate lateral skin incision was made (Figure 2d). Although reduction was already achievable, the release was performed to decrease lateral soft-tissue tension before medial stabilization.

The femoropatellar joint was lavaged intra-articularly with 150 mL of normal saline, delivered in three 50 mL syringe aliquots. The medial arthrotomy was closed by capsular imbrication using United States Pharmacopeia (USP) size 1 polydioxanone in a vest-over-pants suture pattern (Figure 2e). An additional simple continuous suture line using USP 1 polydioxanone was placed to reinforce the capsular closure.

Following capsular imbrication, a 200 × 200 mm polypropylene mesh (POLYNIAR-SPNS5, ISP Medics Co., Ltd., Wonju-si, Gangwon-do, Republic of Korea) was trimmed to cover the reconstructed medial soft tissues. According to the manufacturer’s product information, this was a woven polypropylene mesh intended for hernia repair. According to the manufacturer’s instructions, the mesh had an average areal weight of 44 g/m^2^ and an average pore size of 0.9 × 0.9 mm. The medial reconstruction measured approximately 8 cm in length. The mesh was trimmed to approximately 10 × 3 cm, providing approximately 1 cm of overlap beyond the proximal and distal margins of the reconstruction. The mesh was positioned extra-articularly over the medial capsular repair and secured to the underlying soft tissues using USP 2-0 polydioxanone in a simple interrupted pattern at approximately 1–2 cm intervals (Figure 2f).

Following mesh fixation, the stifle was repeatedly flexed and extended. The patella tracked within the femoral trochlear groove without evidence of lateral reluxation. No gross trochlear deformity was identified on radiographs or intraoperative inspection, and trochleoplasty was not performed. No tibial tuberosity transposition or other osseous corrective procedure was undertaken.

The subcutaneous tissues were closed using USP 3-0 polydioxanone in a simple continuous pattern, and the skin was closed using USP 3-0 nylon in a simple interrupted pattern. A postoperative bandage was applied. The surgical duration, defined as the interval from skin incision to completion of wound closure, was approximately 65 min.

### 2.4. Postoperative Care and Outcome

The surgical site was covered with sterile gauze, cotton bandage, and an outer elastic bandage. A modified Robert Jones-type bandage was applied to cover the wound and minimize slippage, with the purpose of wound protection rather than rigid stifle immobilization. The bandage was changed daily for 10 days, and the pony was maintained on complete stall rest until discharge. Flunixin meglumine was administered at 1.1 mg/kg intramuscularly every 24 h for 10 days. No additional postoperative analgesic medication was administered. No clinically apparent adverse drug reactions were observed during hospitalization.

For surgical site infection prophylaxis, a long-acting antimicrobial preparation containing dihydrostreptomycin sulfate, benzathine penicillin G, and procaine penicillin G (Eagle Long-PS, Eaglevet) was administered at 5 mL intramuscularly every 48 h for four treatments. Each treatment provided approximately 13.3 mg/kg dihydrostreptomycin sulfate, 6667 IU/kg benzathine penicillin G, and 10,000 IU/kg procaine penicillin G.

Caudocranial, lateromedial, and skyline radiographs obtained 2 days postoperatively demonstrated maintenance of patellar reduction (Figure 1d–f). During the early postoperative period, the pony remained markedly reluctant to bear weight on the operated limb. Intermittent partial weight bearing was first observed at approximately postoperative day 10, although ambulation remained substantially impaired. Gait assessments during hospitalization were limited to walking; no trot assessment was performed. No stifle swelling or increased local temperature was observed during the period of impaired weight bearing. Synovial fluid analysis and bacterial culture were not performed.

No surgical site infection, wound dehiscence, seroma formation, or clinical evidence of patellar reluxation was observed during hospitalization. The pony was discharged on postoperative day 12. No specific post-discharge exercise restriction was prescribed, but the owner was advised to avoid particularly uneven or difficult terrain. At telephone follow-up 37 days after surgery, the owner reported that the pony was ambulatory and walking more frequently. An owner-provided video recorded on 23 October 2026 (postoperative day 130; Video S2) showed no overt lameness during walking. No obvious gait abnormality of the left hindlimb was apparent in the same footage. The footage did not include trotting. Return to the hospital for direct examination and repeat radiography was not feasible because of the owner’s transportation constraints. No direct clinical examination was performed after discharge, and radiographic follow-up remained limited to postoperative day 2. The clinical course and available follow-up are summarized in Table 1.

## 3. Discussion

This case describes mesh-augmented medial soft-tissue reconstruction in an adult Shetland pony with unilateral lateral patellar luxation of presumed traumatic origin and no gross osseous deformity.

Kang et al. [3] reported bilateral lateral patellar luxation in a neonatal Shetland pony with hemorrhage and disruption of the medial periarticular tissues. In the present case, the inciting event was not witnessed and no gross traumatic rupture was identified. Acute onset and environmental circumstances supported the classification of the luxation as being of presumed traumatic origin.

An underlying developmental predisposition remains possible despite the owner-reported absence of previous gait abnormalities. Lateral patellar luxation with trochlear ridge hypoplasia has been reported in Miniature Horses [6], and a breeding study in Shetland ponies supported a hereditary component to congenital lateral patellar (sub)luxation [7].

Reduction became possible after anesthesia and surgical exposure, suggesting that relaxation and altered periarticular tension may have facilitated repositioning. The patella remained reduced during passive flexion and extension before lateral release. Nevertheless, the surgeon elected to perform lateral release, medial capsular imbrication, and mesh augmentation because of concern about recurrent luxation during subsequent weight bearing after the preceding period of persistent luxation. This decision reflected clinical judgment rather than demonstrated instability after reduction.

Differences in stifle flexion and radiographic projection may explain the apparent slight cranial positioning of the distal femur relative to the tibia on the preoperative lateromedial radiograph. The intraoperative flexion–extension assessments evaluated patellar tracking and reluxation rather than femorotibial stability.

The absence of gross trochlear deformity on radiographs and intraoperative inspection supported a soft-tissue approach without trochleoplasty, as described in the traumatic case reported by Kang et al. [3].

### 3.1. Polypropylene Mesh Augmentation

Lateral release and medial imbrication are established approaches to lateral patellar luxation [3,8]. Mesh reinforcement has been described in an Arabian foal with congenital luxation [5] and in a foal treated with additional block-recession trochleoplasty [1]. The present report applies this existing augmentation approach to an adult pony without gross osseous deformity. The principal clinical and surgical differences are summarized in Table 2.

Polypropylene mesh has also been used to reconstruct ruptured collateral ligaments of the equine metatarsophalangeal joint [9]. In the present pony, the mesh was intended to reinforce the medial capsular repair. Selection of this product was informed by the manufacturer’s reported biocompatibility testing, including implantation, cytotoxicity, and maximization sensitization tests conducted by the Korea Testing Laboratory (KTL).

Absorbable polydioxanone sutures were selected for initial fixation of the mesh to the underlying soft tissues, with the expectation that tissue healing and fibrous incorporation would contribute to continued fixation as the sutures lost tensile strength.

Potential delayed mesh-related complications include chronic infection, draining sinus formation, and a subsequent need for mesh removal. A retrospective study of equine incisional hernia repair reported more short- and long-term complications in horses receiving mesh support [10]. These observations concern abdominal wall reconstruction and cannot be used to estimate complication rates for extra-articular stifle augmentation. In the patellar repair reported by Gustafsson et al. [5], wound dehiscence, a medial subcutaneous abscess, and femoropatellar sepsis occurred, although the authors did not consider the incorporated mesh to be associated with the infection. These reports highlight the importance of postoperative surveillance.

### 3.2. Postoperative Outcome

Radiographs confirmed patellar reduction on postoperative day 2, and no clinical evidence of reluxation or surgical site complications was observed during hospitalization. Recovery of weight bearing was slower than in the neonatal pony reported by Kang et al. [3], but differences in age, preoperative course, and management preclude direct comparison of surgical efficacy.

Postoperative pain, increased soft-tissue tension following capsular imbrication, periarticular contracture, or postoperative synovitis may have contributed to the delayed recovery of weight bearing.

Flunixin treatment was extended beyond the manufacturer-recommended 5 days to a total of 10 days to maintain postoperative analgesia. Intramuscular flunixin administration has been associated with clostridial myonecrosis in horses [11].

The owner-provided video recorded on postoperative day 130 showed improved ambulatory function, with no overt lameness apparent during walking (Video S2).

### 3.3. Limitations

The main diagnostic limitations were the unwitnessed inciting event, qualitative assessment of trochlear morphology, and absence of contralateral radiographs, ultrasonography, and advanced imaging. These limited characterization of subtle morphological abnormalities, developmental predisposition, and periarticular injury. Additional imaging of the pelvis and coxofemoral joint was not performed. Cartilage assessment was gross and was not documented in dedicated photographs. Femorotibial instability and resistance to medial patellar displacement after lateral release were not specifically assessed. The causes of delayed weight bearing were not objectively established, and septic arthritis could not be definitively excluded because synovial fluid analysis and bacterial culture were not performed.

Standardized serial lameness scores, objective gait analysis, and kinetic assessment were unavailable. Inpatient assessments were limited to walking, and post-discharge follow-up comprised a telephone report and owner-provided walking footage without a trot assessment. No lameness grade was assigned using the American Association of Equine Practitioners (AAEP) scale at either remote follow-up. No direct examination was performed after discharge, and radiographic follow-up remained limited to postoperative day 2. Consequently, sustained patellar reduction, femorotibial stability, complete functional recovery, and delayed mesh-associated complications could not be assessed adequately. The post-discharge exercise instructions were not a validated rehabilitation protocol.

Finally, the single-case design and absence of biomechanical testing or a comparator treated without mesh prevent assessment of the independent contribution of mesh augmentation or the individual effects of the combined procedures. Mesh incorporation and long-term fixation were not directly assessed, and the manufacturer-reported biocompatibility testing does not establish safety or efficacy for equine stifle implantation. Comparative studies with standardized clinical assessment and longer imaging follow-up are required to determine the clinical value of this approach.

## 4. Conclusions

Soft-tissue reconstruction with polypropylene mesh augmentation was technically feasible in this adult Shetland pony with lateral patellar luxation of presumed traumatic origin. Reduction was confirmed radiographically on postoperative day 2. Recovery of weight bearing was initially slow, but no overt lameness was apparent during walking in owner-provided footage recorded on postoperative day 130.

## Figures and Tables

**Table 1:** Timeline of clinical course and available follow-up.

Date/Time Point	Event and Available Findings
Evening of 31 May 2026	Suspected inciting event; not witnessed.
1 June 2026	Owner first observed near non-weight-bearing right hindlimb lameness.
15 June 2026; POD 0	Referral examination, diagnostic imaging, and surgery.
POD 2	Radiographs confirmed maintenance of patellar reduction.
Approximately POD 10	Intermittent partial weight bearing was first observed.
POD 12	Discharge.
POD 37	Telephone follow-up: the owner reported more frequent walking.
23 October 2026; POD 130	Owner-provided walking video showed no overt lameness; no trot assessment, direct clinical examination, or repeat radiography was available.

POD, postoperative day.

**Table 2:** Clinical and surgical comparison of the present case with the reports by Kang et al. [3] and Gustafsson et al. [5].

Characteristic	Kang et al. [3]	Gustafsson et al. [5]	Present Case
Patient	8-day-old female Shetland pony	3 h-old, 40 kg Arabian filly	3-year-old, 75 kg female Shetland pony
Laterality and etiology	Bilateral; traumatic	Left; congenital	Right; presumed traumatic
Patellar reduction	Gentle traction during surgery	Initially reducible; irreducible before surgery	Manually reducible after anesthesia and surgical exposure, before lateral release
Trochlear findings	Radiographically normal	Radiographically normal	No gross radiographic or intraoperative deformity
Medial soft tissues	Ligament, fascia, and capsule damage with hemorrhage	No traumatic disruption reported	No gross traumatic rupture identified
Lateral release	Performed	Performed	Retinaculum and fascia; medial incision with lateral skin retraction
Medial reconstruction	Six vertical mattress sutures; 2-0 PDS; quadriceps fascia incorporated	Seven vertical mattress sutures; USP 0 PDS; capsule, sartorius tendon, and medial patellar ligament	Medial capsule: USP 1 PDS vest-over-pants imbrication with an additional simple continuous suture line
Mesh augmentation	None	3 × 7 cm polypropylene mesh; USP 0 PDS interrupted	10 × 3 cm polypropylene mesh over an approximately 8 cm repair, with 1 cm overlap at each end; USP 2-0 PDS simple interrupted sutures
Osseous procedure	None	None	None
Reported outcome	Normal walking by POD 3; running by POD 5	Sound at walk; occasional trot lameness at 10 months after discharge (see note)	Partial weight bearing from approximately POD 10; more walking reported at POD 37; no overt walk lameness in owner video at POD 130

Outcomes reflect different follow-up methods and durations and are not directly comparable. For Gustafsson et al. [5], the Outcome Section specifies 10 months after discharge, whereas the abstract specifies 10 months after surgery; the table follows the Outcome Section. PDS, polydioxanone; POD, postoperative day; USP, United States Pharmacopeia.

## Data Availability

The anonymized clinical data supporting the findings of this case report are available from the corresponding author upon reasonable request, subject to institutional policies and the scope of the owner’s consent.

## Supplementary Materials

The following supporting information can be downloaded at: https://www.mdpi.com/article/10.3390/ani16213501/s1, Video S1: Preoperative gait during walking in a Shetland pony with right lateral patellar luxation; Video S2: Owner-recorded gait during walking on postoperative day 130 in the same Shetland pony.

## Abbreviations

The following abbreviations are used in this manuscript:

IV	Intravenous
IM	Intramuscular
PDS	Polydioxanone
POD	Postoperative day
USP	United States Pharmacopeia
AAEP	American Association of Equine Practitioners
